# Rate-Determining Step for Electrochemical Reduction of Carbon Dioxide into Carbon Monoxide at Silver Electrodes

**DOI:** 10.1021/acscatal.4c00192

**Published:** 2024-05-15

**Authors:** Etienne Boutin, Sophia Haussener

**Affiliations:** Laboratory of Renewable Energy Science and Engineering, École Polytechnique Fédérale de Lausanne, Station 9, 1015 Lausanne, Switzerland

**Keywords:** Electrochemistry, Carbon Dioxide Reduction, Carbon Monoxide, Silver, Mechanism, Rate-Determining Step

## Abstract

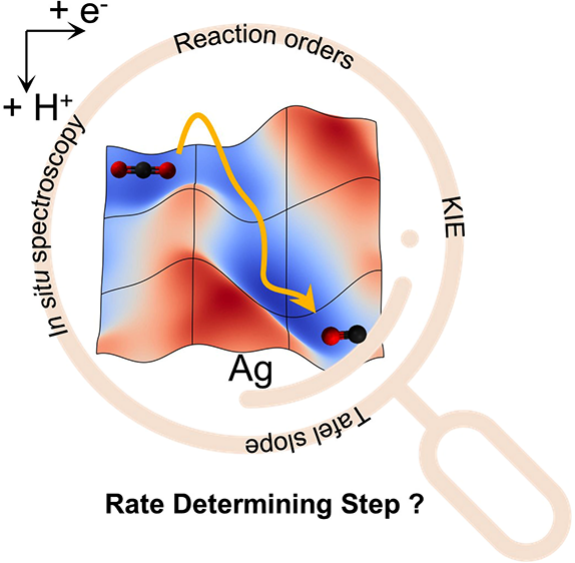

Silver is one of the most studied electrode materials for the electrochemical reduction of carbon dioxide into carbon monoxide, a product with many industrial applications. There is a growing number of reports in which silver is implemented in gas diffusion electrodes as part of a large-scale device to develop commercially relevant technology. Electrochemical models are expected to guide the design and operation toward cost-efficient devices. Despite decades of investigations, there are still uncertainties in the way this reaction should be modeled due to the absence of scientific consensus regarding the reaction mechanism and the nature of the rate-determining step. We review previously reported studies to draw converging conclusions on the value of the Tafel slope and existing species at the electrode surface. We also list conflicting experimental observations and provide leads to tackling these remaining questions.

## Introduction

CO_2_ electrochemical reduction (CO_2_ER) at silver (Ag) electrodes in an aqueous environment is a well studied reaction.^1^ This metal, along with gold (Au), has the specificity to be highly selective for CO_2_ reduction to carbon monoxide (CO) over the competing hydrogen evolution reaction (HER) that involves water reduction into hydrogen (H_2_).^2^ The most promising devices toward applications usually feature a gas diffusion electrode (GDE) for gaseous CO_2_ supply to the catalyst. The development of a mathematical model that correlates device performances with operating conditions would provide an enormous contribution toward system optimization. Nevertheless, in GDE conditions, the number of phenomena still under investigation remains too high for the development of a truly predictive model. The spacial distribution of electrolyte, gaseous reactant, produced bubbles, charge carrier in the membrane, and electrochemical surface area (ECSA) under operational conditions remains to be clarified.^3^ Moreover, the exact mechanism of the reaction and the nature of its rate-determining step (RDS) are not yet the object of a consensus, even when investigated in much simpler conditions. In this Review, we will gather precedent experimental determination of the Tafel slope, reaction order for CO_2_, bicarbonate (HCO_3_^–^), and aqueous proton (H_aq_^+^) as well as results from kinetic isotope effect (KIE) experiments where protons are replaced by deuterium cation (D^+^). We compare these results with *in situ* infrared (IR) and Raman investigations at Ag electrodes under (or approaching) CO_2_ER conditions and highlight both self-consistent observations and conflicting observations. From this unified view, we will draw conclusions on the mechanism and the nature of the RDS and will identify unexplained behavior that must be rationalized in priority to complete our understanding of the CO_2_ER at Ag electrodes.

## Mechanism

**Figure 1:**
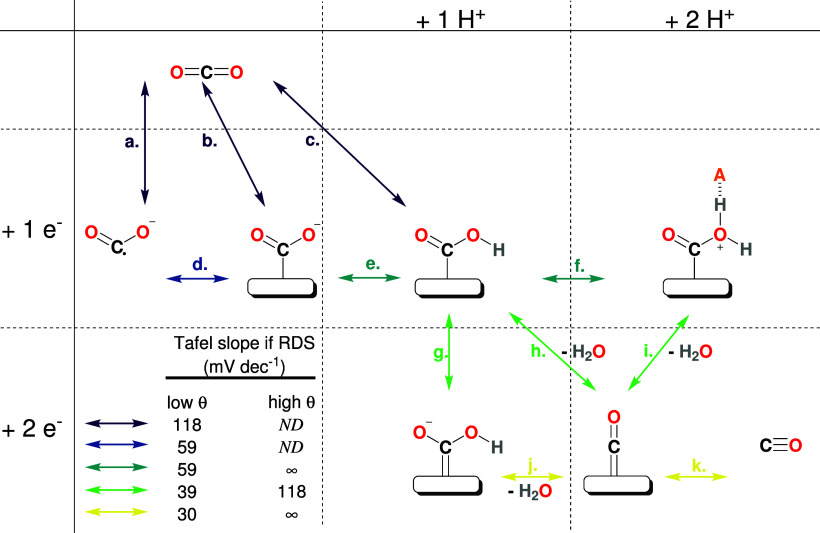
Previously reported mechanisms for CO_2_ER on a Ag electrode in aqueous electrolyte. Different columns are associated with proton (H^+^) transfer, while rows are associated with electron transfer. Here, H^+^ is a generic term that can refer to proton addition by free H_aq_^+^, H_2_O, HCO_3_^–^, or any other present acid (referred to as AH^+^ in this picture). The H^+^ step can also be understood as a CO_2_ addition, acting as a Lewis acid rather than a H^+^ addition. It can also be understood as an OH^–^ removal step without protons being involved (see text). The arrow color refers to the expected Tafel slope value if this step is the RDS, in absence of mass-transport limitations, and if α has the canonical value of 0.5. ND: Not defined.

The mechanism for CO_2_ electrochemical reduction into CO at the Ag electrode has been broadly discussed in the literature.^1,4−11^ Nevertheless, the exact pathway is not fully validated. Figure 1 represents all of the pathways previously reported. The starting reactant is usually accepted to be solvated CO_2,aq_ and not its hydrated forms such as carbonic acid (H_2_CO_3_), bicarbonate (HCO_3_^–^), or carbonate (CO_3_^2–^) that have been discarded by previous experimental studies.^6,12^ It is also accepted since early work^4^ that the reaction cannot be initiated by the outer sphere reduction of CO_2_ into its aqueous CO_2_ radical anion (CO_2,aq_^•–^) (step a) since it is strongly unfavored by thermodynamics (*E*_CO_2_/CO_2,aq_^•–^_^°^ = −1.90 V *vs* SHE).^13^ Such a pathway is expected to happen only at really low potentials, on metals having a high overpotential for HER.^14^ It usually forms mainly formate (HCOO^–^) after fast water deprotonation by CO_2,aq_^•–^ and does not involve the adsorption of reaction intermediates. On Ag, the initiation is instead done by CO_2,aq_ reductive adsorption into COO_ads_^–^ (step b).^7,10^ This step is expected to be followed by protonation of the adsorbate to form protonated adsorbed carbon dioxide anion (COOH_ads_) species (step e).^4,7,10^ In various reports, this protonation step has been considered to be concomitant with the CO_2_ reductive adsorption, directly yielding COOH_ads_ upon the CO_2_ reduction (step c).^1,6,7^ This concomitant initiation step has been preferred due to the high energy of COO_ads_^–^ intermediate computed by density functional theory (DFT) in one study.^6^ In another study, the justification was brought by *in situ* IR observation of COOH_ads_ intermediates at low overpotential switching to COO_ads_^–^ observation at higher overpotential.^9^ Formation of COOH_ads_ intermediates has also been envisioned as a result of adsorption of CO_2_ without prior reduction followed by reductive hydrogenation by an Ag–H intermediate.^8^ But this possibility has been ruled out within the same study as being in contradiction with theoretical and experimental results. Alternative pathways involving the dissociation of COO_ads_^–^ into adsorbed carbon monoxide (CO_ads_) and an adsorbed oxygen anion (O_ads_^–^) have also been proposed in earlier work^15^ but have been abandoned in later literature and are not represented in Figure 1. Rather, the C–O bond cleavage is proposed to happen through further reduction and protonation of the COOH_ads_ intermediates to yield one molecule of water and one CO_ads_. These proton and electron transfer steps have been proposed to happen in different orders, either protonation followed by reduction (steps f + i) or reduction followed by protonation (steps g + j) or concomitantly (step h).^7,10^ Eventually, the CO_ads_ desorbs in a simple chemical step (step k).^7,10^ For the protonation of the COOH_ads_ intermediate, the protonation is sometimes expected on the second oxygen, yielding C(OH)_2,ads_ after subsequent reduction,^16^ or a COOH_ads_ intermediate is expected to act as a Brønsted base, releasing a hydroxide (OH^–^) rather than being protonated before water removal.^4^

## Rate Limiting Steps

The mechanisms depicted in Figure 1 involve many pathways and steps, requiring access to many kinetic constants for a complete model, even if the exact pathway were identified. In CO_2_ER conditions, the applied potential is usually away from equilibrium (overpotential larger than 120 mV) so that the thermodynamics of the overall reaction are mainly downhill. In the absence of mass-transport limitations, the rate of the reaction will be controlled by kinetic barriers. In most cases, only one step, significantly slower than all the other, determines the rate of the whole reaction. From a modeling point of view, the identification of this RDS has important implications as it reduced significantly the model complexity. It also allows a proper mass weight of the kinetic expression with the reactants specifically involved in the RDS. Unfortunately, for simple reactions such as CO_2_ER at Ag electrodes, there is still controversy on the nature of the RDS. It has been successively identified as step b,^11,12^ step c,^6^ step e,^10^ or step k.^17^ In other studies, it has been suggested that the nature of the RDS is a function of the Ag structure, going from step b or c at flat Ag electrodes to step e at porous or nanostructured Ag.^6,10^ In another study, it is suggested that, at porous or nanostructured Ag, the RDS is not step e but HCO_3_^–^ migration inside the pores, which means mass-transport limited without intervention of a kinetic RDS. It should be noted that, in some cases, the justification for the nature of the RDS relies on experiments performed in a nonaqueous solvent and at an electrode different than Ag such as inert mercury (Hg) electrode.^12^ Hereafter, we describe the experimental observations that were used to assign one step as the RDS and comment on whether they agree with each other.

One of the most used pieces of information to assign the RDS is the value of the Tafel slope. The derivation of the Tafel equation for a two-electron, two-proton process such as CO_2_ER at a Ag electrode implies a series of starting hypotheses that we describe hereafter. Because steps following the RDS are assumed as much faster, the concentration of surface intermediates following this step is approximated as zero and the reverse reaction of the RDS is neglected. In these conditions, the current density expressions reduce to the forms below depending on whether the RDS is a chemical step (no electron transfer involved, eq 1) or an electrochemical step (involving an electron transfer, eq 2).
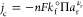
1
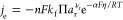
2

In the equations above, c and e subscripts stand for the nature of the RDS, *chemical* or *electrochemical*, respectively. The letter *n* corresponds to the number of electron transferred in the overall reaction (two in CO_2_ to CO conversion), *F* is the Faraday constant (96,485 C mol^–1^), and *k*_f_^°^ is the standard forward rate constant associated with the RDS and is expressed in mol s^–1^ cm^–2^. The term Π*a*_r_^ν^ is the activity product of all reactants involved in the step of interest, each raised to the power of ν_r_, which is the reaction order of the reactant. In modeling, usually the reaction order is assumed to equal the stoichiometric coefficient for the step of interest and is written in its simplest form (when the sum of all reactant stoichiometric coefficients is equal to the molecularity of the step). Here we are interested in the forward step only, and thus, product concentrations of the step are not part of the expression. It is also important to note that when a step is an adsorption step, the activity of the free active site should be included in the activity product. The activity of the active site is usually considered equal to the ratio of free active sites θ *= . When the step is electrochemical, the rate constant becomes potential dependent and *k*_f_^°^ becomes . The term α is the *charge transfer coefficient* (unitless and ranging from 0 to 1), *R* is the ideal gas constant (8.314 J K^–1^ mol^–1^), and *T* is the temperature in Kelvin. The term η is the overpotential, defined as *E*_app_ – *E*° where *E*_app_ is the applied potential at the electrode and *E*° is *E*_CO_2_/CO_^°^, the standard potential for the overall reaction. It should be noted that, for electrochemical steps, we are not using the *standard* rate constant *k*_f_^°^, but *k*_f_ that we refer to as the *apparent* rate constant. The use of the *standard* rate constant in the electrochemical term must be associated with the overpotential of the specific step, which requires access to the value of *E*_step_^°^. In the absence of this value, we use the overpotential of the overall reaction, which, after rearrangement, results in eq 2, where *k*_f_ is equal to  and the standard potential of the specific step is hidden inside the value of the apparent forward rate.

For electrochemical steps, −*nFk*_f_ is usually replaced by the term *j*_0_ called the *exchange current density* in reference to the Butler–Volmer theory.^18^

When the RDS is preceded by other steps, they are considered at equilibrium since they proceed much faster than the RDS. In the case the current is too small to induce a concentration gradient near the surface, the activity of the surface intermediate (usually taken as the ratio of coverage) can be expressed *via* the usual equilibrium constant for a chemical step (eq 3) or the Nernst equation (eq 4).^18^ Because these steps are in equilibrium, the term *a*_*i*_^ν_*i*_^ now includes the activity of all species *i* involved in the step (reactants and products) raised to the power of ν_*i*_. The term *n* refers to the number of electrons transferred in the step of interest.
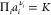
3
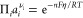
4

**Figure 2:**
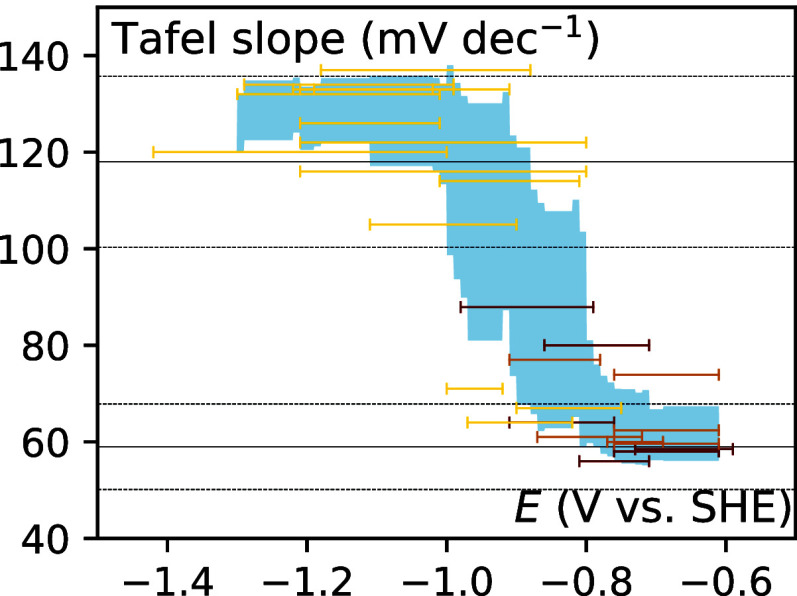
Reported values for CO_2_ER to CO Tafel slopes at a Ag electrode as a function of potential in SHE scale. The color of the bar indicates the type of Ag structure, being sorted into flat polycrystalline (yellow), porous (light brown), or nanostructured (dark brown). Plain lines indicate the position of the canonical values (59 and 118 mV dec^–1^), and dashed lines, the 15% deviation from these values. The blue shaded region indicates the average reported value within standard deviation. Experimental details and references are compiled in Table S1.

If several steps are in equilibrium prior to the RDS, their expression can be combined with the RDS expression to obtain a more compact expression where surface intermediate concentration has been canceled and all potential dependent terms are merged into a single exponential. Such a derivation has been presented extensively for all possible RDS in recent literature.^11,19^ The exponential term of this compact expression is dependent on the step and is the basis of the Tafel slope analysis. In this method, the assumption is taken that the *charge transfer coefficient α* has the theoretical value of 0.5, which has important implications for the rest of the analysis. This term expresses the proportion of a change in applied potential that will be translated into a change of the energy barrier for the associate electron transfer. It can vary theoretically between 0 and 1. In practice, it is almost always measured to be between 0.3 and 0.7 and Bard suggests the 0.5 value might be considered in the absence of actual measurements.^18^ This value being fixed, the slope of the plot log_10_(*i*) *vsE* expressed in mV decade^–1^, is a function of the RDS.^11,19^ In Figure 1, the color of each step indicates what Tafel slope value would be expected if the step were the RDS. Because the potential will also influence the concentration of the surface intermediate, the slope is expected to change at sufficiently low potential when intermediate coverage reaches saturation. The slope expected at high coverage is also displayed in Figure 1. The Tafel slope analysis is only valid when the mass-weight term in the pre-exponential is not interfering with the slope value. To avoid mass-transfer effects, the analysis is usually done in a region of low current, where effects of concentration are small. Another method consists in computing the surface concentration of the species involved in the kinetic term and correcting the current from the mass weight to only keep the variation of the exponential term as a function of potential.^1,20^ We believe such a correction should be done systematically since, even at low current, proton concentration may deviate when there is not a buffer electrolyte maintaining the pH value close to the bulk pH value. At polycrystalline Ag electrodes, a Tafel slope near 118 mV dec^–1^ has been observed by several groups, pointing toward step b or c being the RDS.^1,5,7,11,20−24^ But for porous or nanostructured Ag electrodes, there have been many reports of the Tafel slope being near 59 mV dec^–1^,^5−7,10,21−24^ suggesting a different RDS (namely step e) when Ag has higher rugosity/porosity. Nevertheless, these two types of Ag structures were not tested under identical conditions for practical reasons. As already mentioned, the current range where the Tafel slope analysis is possible is where the current is small enough not to induce concentration gradients (typically below 0.5 mA cm^–2^). For the case of the CO_2_ER, the current should also be sufficient so that gas products can be detected and distinguished between current density for CO (*j*_CO_) and current density for H_2_ (*j*_H_2__). Because current is much more important for porous/nanostructured Ag than for flat polycrystalline Ag, Tafel slopes for the two types where not obtained in the same potential range. This has been addressed by Dunwell et al. that constructed a 10.5 cm^2^ polycrystalline Ag electrode in order to access the Tafel slope for bulk Ag at less negative potential.^19^ They observed that the Tafel slope for bulk Ag is also 67 mV dec^–1^ at a low overpotential. They conclude that any observation at a more negative potential is influenced by mass transport and that the RDS for the CO_2_ER at Ag electrodes should be step e at all potentials. This interpretation is not likely as the Tafel slope of 118 mV dec^–1^ previously reported was determined at current density sometimes below 0.1 mA cm^–2^ where effects of concentration should still be residual. To get a better picture, we gather 29 Tafel slopes reported from 12 independent studies and plot the value against a reversible hydrogen electrode (RHE) (Figure S1) and standard hydrogen electrode (SHE) (Figure 2). It appears from the plots that, independent of the Ag structure, there is a shift in Tafel slope values between 59 and 118 mV dec^–1^ at around −0.5 V *vs* RHE. This indicates that the mechanism of the reaction and the nature of the RDS is not affected by the structure of Ag, unlike previously suggested. Nevertheless, the explanation for this slope change remains open. There is not a single RDS for which the slope change associated with intermediates saturating the surface ranges from 59 to 118 mV dec^–1^. The slope change could indicate that the RDS is changing with potential. In this case, step e or f is at low overpotential and then step b or c is at more negative potential. Or it could be step e or f followed by step g, h, or i if the intermediates’ coverage becomes significant. Interestingly, when plotting the Tafel slope ranges *vs* the SHE electrode, the transition is less sharp than *vs* RHE with a much broader overlapping region (*ca*. 300 mV instead of 100 mV), suggesting that the RDS transition is pH dependent. It is worth noting that a similar change in the Tafel slope has also been observed at the Au electrode.^25^ Since Ag and Au electrodes share similar reactivity toward CO_2_ER, it could be that the same mechanism and same RDS changes are at play for both metals.^2^

It should be emphasized that the Tafel slope analysis relies on the assumption that α is equal to (or near) 0.5. This assumption is not yet validated for CO_2_ER on Ag, since Bayesian analysis has revealed that the cardinal value of 0.5 is not more likely than other values when the data points selected for the Tafel slope determination are liberated from operator intervention.^26^ Nevertheless, as stated above, its value is most likely constrained between 0.3 and 0.7. Deviation from the theoretical value may also arise during Tafel slope analysis if more than one reaction is occurring at the surface. The intermediate coverage of one could modify the current response of the other, which is the case here with both CO and H_2_ being produced. But because HER is also expected to be limited by the proton reductive adsorption,^12^ Ag–H intermediates are not expected to populate the Ag surface, which is confirmed by *in situ* spectroscopy (see later).

We have shown that the Tafel slope allows creation of a short list of possible RDSs but is not sufficient, as a single value can relate to several steps.^11,19^ The Tafel analysis needs to be complemented by other observations. There have been several attempts to determine the reaction order for CO_2_, aqueous proton (H_aq_^+^), or HCO_3_^–^, which should provide an indication of the nature of the RDS. The reaction order of CO_2_ have been determined to be 1.14 at −0.2 V *vs* RHE,^27^ 0.942 at −0.35 V *vs* RHE,^5^ 0.5 ± 0.02 at −0.35, −0.45, and −0.80 V *vs* RHE,^6^ and 1.56 at −1.0 V *vs* RHE.^8^ The discrepancy between these values is likely caused by all these experiments being carried out at a fixed RHE potential, which means at different overpotentials since the CO_2_ concentration affects the pH. In the derivation of the rate equation, η is defined as *E*_app_ – *E*_CO_2_/CO_^°^, the applied potential *vs* the *standard* potential, and is thus independent of pH, its influence being reflected in the pre-exponential term when required.^11^ Correction of the collected data from the RHE scale to the SHE scale is possible provided the Tafel slope is known but creates a risk of misinterpretation as different Tafel slope values result in different CO_2_ reaction orders, as demonstrated on Au by Dunwell et al.^19^ The same applies to HCO_3_^–^ order, which has been determined to be 0.03 at −0.6 V *vs* RHE,^7^ 0.53, 0.51, and 0.50 at −0.8, −0.45, and −0.387 V *vs* RHE, respectively,^6^ and 0.8 at −0.4 V *vs* RHE.^10^ In all these experiments, different HCO_3_^–^ concentrations resulted in different overpotentials at fixed potential *vs* RHE, as pH is affected by the HCO_3_^–^ concentration at constant *p*_CO_2__. In one report only, the HCO_3_^–^ order is determined at a fixed potential *vs* silver chloride electrode (Ag/AgCl) and yields an *apparent* reaction order of *ca*. 0 (−0.015 at −1.01 V *vs* Ag/AgCl).^5^ Here we referred to the reaction order as being *apparent* since varying CO_2_ concentrations at fixed HCO_3_^–^ concentration or *vice versa* results in varying H_aq_^+^ concentrations that will be reflected in the rate constant. As an example, if a step is first order in both H_aq_^+^ and HCO_3_^–^, the term [H_aq_^+^][HCO_3_^–^] will appear in the pre-exponential. But because of the acid–base equilibrium of CO_2_ in water (CO_2_ + H_2_O ⇌ H_aq_^+^ + HCO_3_^–^), [H_aq_^+^][HCO_3_^–^] is equal to *K*_CO_2__[CO_2_] and is constant at fixed CO_2_ concentration. The current thus appears as zero order *vs* HCO_3_^–^ concentration. In Table 1, we provide the mass weight associated with each step being the RDS (with the exception of steps a or d, already ruled out above). Because [H_aq_^+^] is proportional to [CO_2_] at fixed HCO_3_^–^ concentration and inversely proportional to [HCO_3_^–^] at fixed CO_2_ concentration, the resulting *apparent* reaction order for CO_2_, HCO_3_^–^, and H_aq_^+^ is provided for each RDS and each possible acid–base reaction in the RDS. When the acid–base reaction is a proton addition, the source of the proton is either H_aq_^+^, HCO_3_^–^, or H_2_O. When the acid–base reaction is associated with a water removal as in steps f + i, h or g + j, the RDS can involve a proton addition as mentioned just above followed by water removal, but it can also be CO_2_ addition, acting as a Lewis acid, followed by HCO_3_^–^ removal or simply OH^–^ removal as a simple chemical step. All of these possibilities are reported in Table 1 and result in different reaction orders. It should be noted that, in this table, we consider in first approximation that the CO_2_ Henry’s constant and acid–base equilibrium constant are not affected by variation in HCO_3_^–^ concentration. For the cases where HCO_3_^–^ is the proton donor in the RDS, the reaction order for H_aq_^+^ cannot be determined experimentally, as it involves changing the electrolyte nature and thus the proton donor nature. It is thus reported as Not Determined (ND) in Table 1. We believe a careful determination of this reaction order would be of great value to confirm a possible step as the RDS for CO_2_ER at the Ag electrode. At the moment, the only available data determined *vs* fixed reference electrode gives a HCO_3_^–^ reaction order of −0.015 at −0.8 V *vs* SHE.^5^ Such a value is consistent with the RDS being step b, or step c or e if water is the proton donor, or step f, h, or j if HCO_3_^–^ is the proton donor. H_aq_^+^ reaction order of zero has also been determined by Deng et al. since they show the CO production is almost independent of pH when reported *vs* SHE.^11^ This narrows down the possible RDS list to step c, or step e associated with water as a proton donor. The reaction order of water is impossible to probe in aqueous solvent, but its acidity can be varied through KIE.^28^ Such experiments, where H_2_O is replaced by D_2_O, have been conducted by Deng et al. and revealed that despite HER being strongly slowed down at the Ag electrode, the CO production is almost insensitive to the weight of the hydrogen atoms.^11^ This experimental fact suggests no proton transfer is involved in the RDS, including a proton transferred by water.

**Table 1:** Expected Mass Weight and *Apparent* Reaction Order for CO_2_, HCO_3_^–^, and H_aq_^+^ Depending on RDS and on the Nature of the Acid–Base Reaction Involved in the RDS, If Any^a^,^b^

**RDS**	**Acid–base reaction of the step**	**Mass weight**	**Apparent CO**_**2**_**reaction order at constant [HCO_3_^–^]**	**Apparent HCO**_**3**_^–^**reaction order at constant *p*_CO_2__**	**Apparent H**_aq_^**+**^**reaction order at constant *p*_CO_2__**
b	x	[CO_2_]	1	0	0
c	+ H_aq_^+^	[CO_2_][H_aq_^+^]	2	–1	1
	+ HCO_3_^–^ – CO_3_^2–^	[CO_2_][HCO_3_^–^]	1	1	ND
	+ H_2_O – OH^–^	[CO_2_]	1	0	0
e	+ H_aq_^+^	[CO_2_][H_aq_^+^]	2	–1	1
	+ HCO_3_^–^ – CO_3_^2–^	[CO_2_][HCO_3_^–^]	1	1	ND
	+ H_2_O – OH^–^	[CO_2_]	1	0	0
f	+ H_aq_^+^	[CO_2_][H_aq_^+^]^2^	3	–2	2
	+ HCO_3_^–^ – CO_3_^2–^	[CO_2_][H_aq_^+^][HCO_3_^–^]	2	0	ND
	+ H_2_O – OH^–^	[CO_2_][H_aq_^+^]	2	–1	1
	+ CO_2_	[CO_2_]^2^[H_aq_^+^]	3	–1	1
g	x	[CO_2_][H_aq_^+^]	2	–1	1
h	+ H_aq_^+^	[CO_2_][H_aq_^+^]^2^	3	–2	2
	+ HCO_3_^–^ – CO_3_^2–^	[CO_2_][H_aq_^+^][HCO_3_^–^]	2	0	ND
	+ H_2_O – OH^–^	[CO_2_][H_aq_^+^]	2	–1	1
	+ CO_2_ – HCO_3_^–^	[CO_2_]^2^[H_aq_^+^]	3	–1	1
	– OH^–^	[CO_2_][H_aq_^+^]	2	–1	1
i	x	[CO_2_][H_aq_^+^]^2^	3	–2	2
j	+ H_aq_^+^	[CO_2_][H_aq_^+^]^2^	3	–2	2
	+ HCO_3_^–^ – CO_3_^2–^	[CO_2_][H_aq_^+^][HCO_3_^–^]	2	0	ND
	+ H_2_O – OH^–^	[CO_2_][H_aq_^+^]	2	–1	1
	+ CO_2_ – HCO_3_^–^	[CO_2_]^2^[H_aq_^+^]	3	–1	1
	– OH^–^	[CO_2_][H_aq_^+^]	2	–1	1
k	x	[CO_2_][H_aq_^+^]^2^	3	–2	2

a Reaction order at constant potential *vs* SHE or any other pH-independent reference electrode. Activities of dissolved species are considered equal to concentration, and *a*_H_2_O_ is considered equal to unity.

b x: No acid–base reaction involved, ND: Not determined.

Another experimental technique for RDS determination is *in situ* spectroscopy. Surface intermediates preceding the RDS are expected to be at equilibrium and should accumulate as potential decreases. They should thus be detectable by spectroscopy techniques that probe the vibration mode of species adsorbed at the electrode surface. This is the case of infrared absorption techniques, such as Infrared Reflection Absorption Spectroscopy (IRRAS), Polarization Modulation Infrared Reflection Absorption Spectroscopy (PM-IRRAS), Subtractively Normalized Interfacial Fourier Transform Infrared Spectroscopy (SNIFTIRS), Attenuated Total Reflection Fourier Transform Infrared Spectroscopy (ATR-FTIR), and Surface Enhanced Infrared Absorption Spectroscopy (SEIRAS). Raman scattering techniques such as Raman Spectroscopy (RS) or Surface Enhanced Raman Spectroscopy (SERS) are also able to detect signals coming from adsorbed species at the surface. Nevertheless, the attribution of the peak is not always straightforward. In Table 2, we gathered the reported peaks observed *via* infrared and Raman techniques at the Ag electrode in conditions relevant for CO_2_ER experiments such as a CO_2_ and a CO atmosphere. Observations under air or inert gas are also reported for the identification of Ag–H, HCO_3_^–^, and CO_3_^2–^ peak positions. It should be noted that the data are reported as graphically presented, so that some peaks may not appear in the table, not because they are not detected, but simply because the spectrum as presented does not cover the full wavelength range.

**Table 2:**
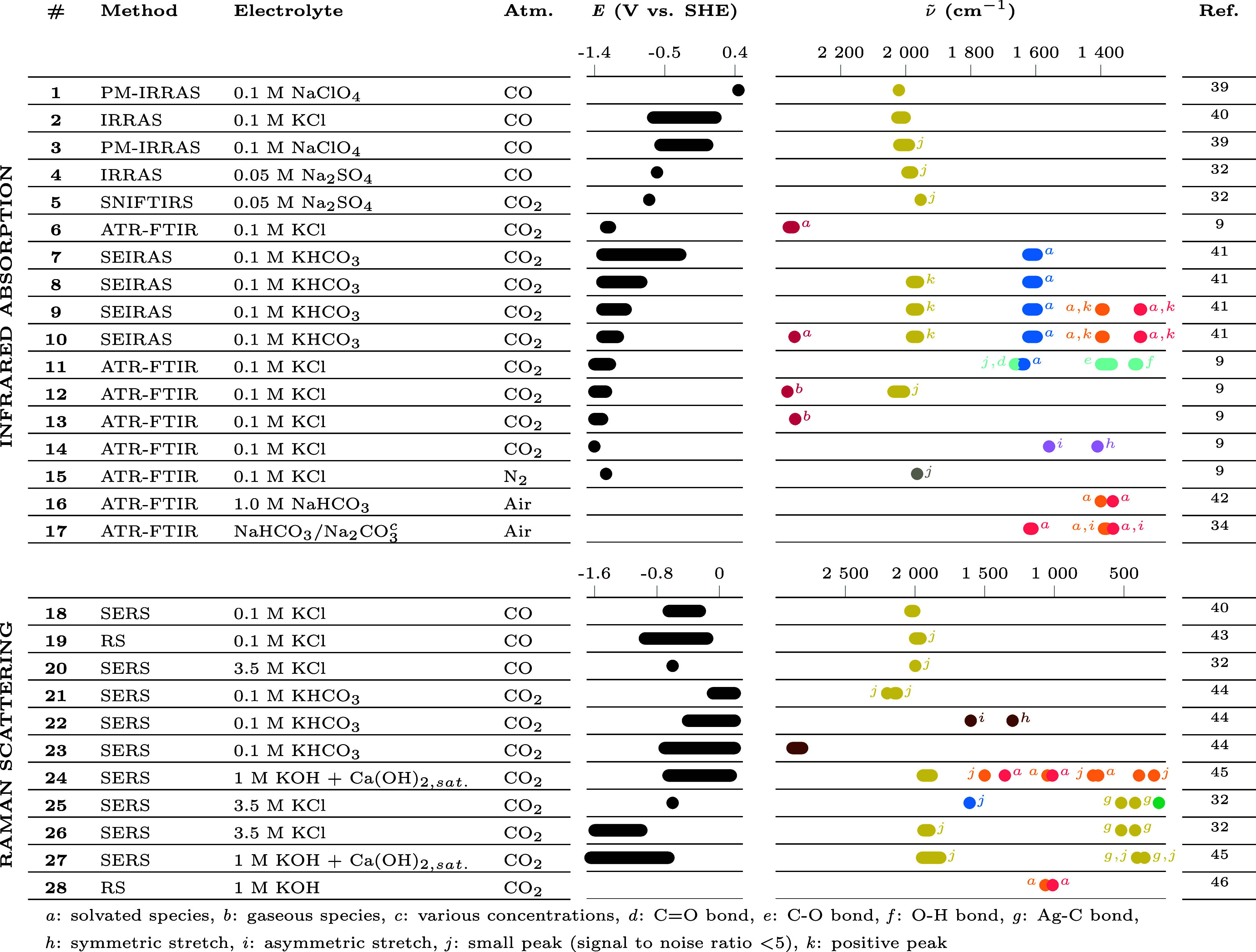
Compilation of Species Observed with *in Situ* IR and Raman Methods at a Ag Cathode during CO_2_ ER Experiments or under Approaching Conditions^I^^9,32,34,39−46^

I Species are reported as attributed in literature: (blue circle) H_2_O, (green circle) Cl^–^, (gray circle) H, (orange circle) CO_3_^2–^, (pink circle) HCO_3_^–^, (red circle) CO_2_, (purple circle) COO^–^, (light blue circle) COOH, (light brown circle) CO, (brown circle) HCOO^–^. Unless otherwise noted, species are considered to be adsorbed at the surface.

One of the important observations is that the vibration signature of Ag–H is observed only once in 25 experiments made at cathodic potentials (only at entry 15 in Table 2). This single observation of the Ag–H peak is performed by ATR-FTIR at −1.45 V *vs* Ag/AgCl in 0.1 M KCl saturated with nitrogen (N_2_) in a spectrum that is particularly noisy.^9^ Also, the identification of the peak at this position (1965 cm^–1^) relies on observations on another metal (Ga–H) and in the gas phase.^29,30^ This converges toward a Ag–H signature being mainly absent in CO_2_ER conditions at the Ag electrode. This observation is in agreement with HER at the Ag electrode being limited by the first electron transfer.^12,31^ It also confirms that Ag–H coverage would be small and is not expected to interfere with *j*_CO_, even at potentials where *j*_H_2__ is non-negligible.

If a step from e to k were the RDS, surface intermediates presented in Figure 1 would accumulate as the potential becomes more negative. Despite many studies in CO_2_ER conditions at the Ag electrode, such intermediates have never been observed with Raman spectroscopy (entries 18 to 28 in Table 2). In only one study with ATR-FTIR,^9^ out of 14 IR experiments (entries 11 and 14 in Table 2), have COO_ads_^–^ and COOH_ads_ been identified. From this observation and the ratio between the COO_ads_^–^ and the COOH_ads_ peak intensities as a function of potential, the authors concluded that the mechanism is going through step c at low overpotential and through steps b + e at higher overpotential. Nevertheless, the attribution of these peaks could be debated. This specific experiment is performed in CO_2_ saturated 0.1 M KCl electrolyte. In this condition, as the reaction goes on, the OH^–^ concentration will build up at the surface and so do HCO_3_^–^ and CO_3_^2–^ concentrations through CO_2_ hydroxylation. It is thus likely that the peaks attributed to COO_ads_^–^ and COOH_ads_ are confused with aqueous HCO_3_^–^ and CO_3_^2–^ species signals. The peaks attributed to COOH_ads_ are at *ca*. 1290 cm^–1^ for the O–H bond, 1380 cm^–1^ for the C–O bond, and 1660 cm^–1^ for the C=O bond. But these peak positions are also matching with dissolved HCO_3_^–^ peaks (1360 and 1620 cm^–1^) and the CO_3_^2–^ peak (1390 cm^–1^) determined independently (entries 16 and 17 in Table 2 and also in precedent literature).^32^ In a later publication from the same group, the assignment is actually changed to aqueous HCO_3_^–^ and CO_3_^2–^ with convincing fits,^33^ even for the peak at *ca*. 1290 cm^–1^ that does not appear when the two aqueous species are probed independently.^34^ The presence of adsorbed intermediates can be mainly ruled out from these observations.

A striking observation is that the peak of adsorbed CO is often reported in CO_2_ER conditions (entries 5, 8–10, 12, 21, and 24–27 in Table 2) and its assignment is consistent with observation of CO adsorption at the Ag electrode under a CO atmosphere (entries 1–4 and 18–20 in Table 2) and CO adsorption at Ag in gas phase.^35,36^ Nevertheless, Ag is expected from DFT calculations to be one of the metals with the weakest affinity for CO.^1,37,38^ In most cases, the peaks near 2000 cm^–1^ are small (with signal-to-noise ratio below 5) while the peaks assigned to Ag–CO located around 500 cm^–1^ observed during one study (entries 25–27 in Table 2) are much clearer. But the assignment of the latter is subject to caution as it falls in the same range as the CO_3_^2–^ peak assigned in another study (entry 24 in Table 2). These observations are pointing toward CO being loosely bound to the Ag electrode or at limited adsorption sites, such as defects as suggested by Oda et al.^32^ This observation would be consistent with a recent study that demonstrated how electrochemical reactions at the Ag electrode are slowed down by the addition of CO.^17^ This observation led the authors to the conclusion that CO_ads_ desorption to CO_aq_ (step k) is determining the rate of CO_2_ reduction at Ag. Nevertheless such a hypothesis should result in a Tafel slope of 30 mV dec^–1^ and the accumulation of CO_ads_ at more negative potentials, which is not consistent with the small peaks detected by *in situ* spectroscopy techniques. This discrepancy remains to be addressed before a complete understanding of the reaction mechanism can be achieved.

It should be also noted that adsorbed species associated with HCOO^–^ have been reported in one study (entry 22 and 23 in Table 2), which is consistent with HCOO^–^ being a minor product of CO_2_ER at the Ag electrode.^1^ Despite its production being slow, the possibility that some intermediates interfere with the CO_2_ to CO mechanism is given. Nevertheless, their presence has been reported up to now only once in the spectroscopy studies we covered.

All the data reported above taken together seem to converge toward the RDS at sufficiently low potential (below −0.6 V *vs* RHE) being the first electron transfer to form the COO_ads_^–^ (step b). This observation is consistent with another recent study where it has been demonstrated that the cations’ presence is essential for the CO_2_ER to happen at the Ag electrodes.^47^ In this study, they suggested that the role of cations is to stabilize the COO_ads_^–^ intermediate *via* electrostatic interaction. They have supported this proposition with consistent DFT calculation. In the same study, the authors have shown that the CO_2_ reduction current grows with cation concentration. This is consistent with the step b rate driving the overall reaction rate. Alternatively, the influence of COO_ads_^–^ stabilization by cations on the current could be through the increase of the intermediates’ coverage if a later step were rate determining. But the lack of spectroscopic signature (see above) seems contradictory with this latter interpretation.

## Conclusion

We reviewed investigations on the mechanism and the RDS for the CO_2_ER at Ag electrodes that have been carried out over decades. We have identified that, for potentials more negative than −0.6 V *vs* RHE, the RDS is likely the reduction of CO_2_ into COO_ads_^–^, in agreement with a Tafel slope value near 120 mV dec^–1^, a zero-order reaction for both H_aq_^+^ and HCO_3_^–^, and the absence of a KIE. However, unexplained behavior remains to be investigated. For example, why does the Tafel slope shift to 60 mV dec^–1^ at potentials less negative than −0.4 V *vs* RHE and what are the corresponding reaction orders of CO_2_, H_aq_^+^, and HCO_3_^–^? Or can CO bind significantly to the surface without its desorption being involved in the RDS? Or is the peak observed in the IR spectroscopy near 1280 cm^–1^ truly associated with solvated CO_3_^2–^ ^32^ or rather HCO_3_^–^, and why does it not appear in experiments conducted with KHCO_3_?^34^ To answer these and other remaining questions, we suggest that reaction order and Tafel slopes are further investigated with the precautions raised in the present Review. Namely, the surface concentration of aqueous species needs to be computed for accurate Tafel slope and reaction order calculations. This requires the use of numerical models associated with sets of data where convection is well controlled.^48^ The surface concentration of adsorbed species also needs to be taken into account by introducing microkinetic models into the analysis. *Ab-initio* calculations could also provide insights into the mechanism and the relative rate of various steps. Nevertheless, care should be taken on the kinetic data since minor errors in energy barriers translate into large errors in kinetic rate due to its exponential form.^49^

## Supporting Information Available

Reported Tafel slopes *vs* SHE scale and compilation of experimental data with references (PDF)
