# Supplementary material for: Rate-Determining Step for Electrochemical Reduction of Carbon Dioxide into Carbon Monoxide at Silver Electrodes
Source: ACS Catal. 2024 May 15;14(11):8437–45. doi: 10.1021/acscatal.4c00192 (PMC11165447; doi:10.1021/acscatal.4c00192)
Supplement: Supplementary file 1 — cs4c00192_si_001.pdf [file cs4c00192_si_001.pdf]

Supporting Information: Rate Determining Step for Electrochemical Reduction of Carbon Dioxide into Carbon Monoxide at Silver Electrodes

Etienne Boutin* and Sophia Haussener*

Laboratory of Renewable Energy Science and Engineering, École Polytechnique Fédérale de Lausanne, Station 9, 1015 Lausanne, Switzerland

E-mail: etienne.boutin@epfl.ch; sophia.haussener@epfl.ch

Phone: +41 21 693 3878

Supplementary Figures

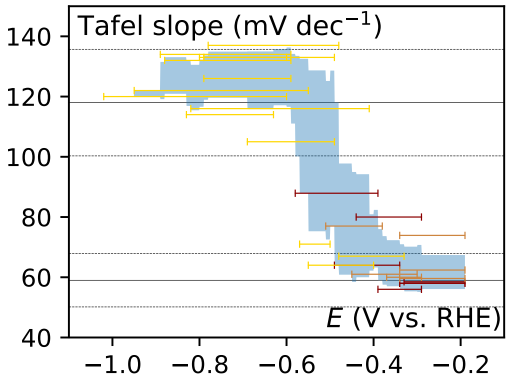

Figure S1: Reported values for CO_2 electrochemical reduction (CO_2ER) to CO Tafel slopes at an silver (Ag) electrode as a function of potential in the reversible hydrogen electrode (RHE) scale. Color of the bar indicates the type of Ag structure, being sorted into flat polycrystalline (yellow), porous (light brown) or nanostructured (dark brown). Plain lines indicate the position of the canonical values (59 and 118 mV dec^{-1}) and the dashed lines the 15% deviation from these values. The blue shaded region indicates the average reported value within standard deviation. Experimental details and references are compiled in Table S1.

Supplementary Tables

Table S1: List of previously reported Tafel slopes at various Ag electrode topologies and in different electrolytes (potassium bicarbonate ($KHCO_3$), sodium bicarbonate ($NaHCO_3$), tripotassium phosphate (K_3PO_4), monopotassium phosphate (KH_2PO_4), or phosphoric acid (H_3PO_4))

Ag type	Electrolyte	*pH*	*E min* vs. RHE	*E max* vs. RHE	*E min* vs. SHE	*E max* vs. SHE	Mass-transport corrected	Tafel slope (mV dec^{-1})	Ref.
Bulk	0.1 M $KHCO_3$	6.8	-0.89	-0.59	-1.29	-0.99	Yes	134	S1
Nano	0.5 M $NaHCO_3$	7.2	-0.49	-0.34	-0.91	-0.76	No	64	S2
Nano	0.5 M $NaHCO_3$	7.2	-0.34	-0.19	-0.76	-0.61	No	58	S2
Bulk	0.5 M $NaHCO_3$	7.2	-0.48	-0.33	-0.90	-0.75	No	67	S3
Bulk	0.1 M $KHCO_3$	6.8	-1.42	-1.09	-1.82	-1.49	Yes	391	S4
Bulk	0.1 M $KHCO_3$	6.8	-1.02	-0.60	-1.42	-1.00	Yes	120	S4
Bulk	0.3 M $KHCO_3$	7.0	-0.69	-0.49	-1.11	-0.90	No	105	S5
Bulk	0.1 M K_3PO_4	6.6	-0.82	-0.41	-1.21	-0.80	No	116	S5
Bulk	0.1 M KH_2PO_4	4.3	-0.95	-0.55	-1.21	-0.80	No	122	S5
Bulk	0.1 M KH_2PO_4 + 0.1 M H_3PO_4	2.9	-0.83	-0.63	-1.01	-0.81	No	114	S5
Bulk	0.1 M $KHCO_3$	6.8	-0.79	-0.59	-1.19	-0.99	No	133	S6
Porous	0.1 M $KHCO_3$	6.8	-0.51	-0.38	-0.91	-0.78	No	77	S6
Porous	0.1 M $KHCO_3$	6.8	-0.37	-0.29	-0.77	-0.69	No	60	S7
Nano	0.5 M $KHCO_3$	7.2	-0.39	-0.29	-0.81	-0.71	No	56	S8
Nano	0.5 M $KHCO_3$	7.2	-0.44	-0.29	-0.86	-0.71	No	80	S8
Bulk	0.5 M $KHCO_3$	7.2	-0.79	-0.59	-1.21	-1.01	No	126	S8
Bulk	0.5 M $KHCO_3$	7.2	-0.88	-0.59	-1.30	-1.01	No	132	S9
Nano	0.5 M $KHCO_3$	7.2	-0.34	-0.19	-0.76	-0.61	No	58	S9
Bulk	0.1 M $KHCO_3$	6.8	-0.78	-0.48	-1.18	-0.88	No	137	S10
Nano	0.1 M $KHCO_3$	6.8	-0.58	-0.39	-0.98	-0.79	No	87.9	S10
Nano	0.1 M $KHCO_3$	6.8	-0.33	-0.19	-0.73	-0.59	No	58.5	S10
Bulk	0.5 M $NaHCO_3$	7.2	-0.80	-0.60	-1.22	-1.02	No	133	S11
Bulk	0.5 M $NaHCO_3$	7.2	-0.57	-0.50	-1.00	-0.92	No	71	S11
Bulk	0.5 M $NaHCO_3$	7.2	-0.55	-0.40	-0.97	-0.82	No	64	S11
Porous	0.5 M $NaHCO_3$	7.2	-0.45	-0.30	-0.87	-0.72	No	61	S11
Bulk	0.5 M $KHCO_3$	7.2	-0.79	-0.49	-1.21	-0.91	No	133	S12
Porous	0.5 M $KHCO_3$	7.2	-0.34	-0.19	-0.76	-0.61	No	73.9	S12
Porous	0.5 M $KHCO_3$	7.2	-0.34	-0.19	-0.76	-0.61	No	62.4	S12
Porous	0.5 M $KHCO_3$	7.2	-0.34	-0.19	-0.76	-0.61	No	59.6	S12

References

(S1) Hatsukade, T.; Kuhl, K. P.; Cave, E. R.; Abram, D. N.; Jaramillo, T. F. Insights into the electrocatalytic reduction of CO_2 on metallic silver surfaces. *16*, 13814–13819, Publisher: The Royal Society of Chemistry.

(S2) Rosen, J.; Hutchings, G. S.; Lu, Q.; Rivera, S.; Zhou, Y.; Vlachos, D. G.; Jiao, F. Mechanistic Insights into the Electrochemical Reduction of CO2 to CO on Nanostructured Ag Surfaces. *5*, 4293–4299, Publisher: American Chemical Society.

(S3) Dunwell, M.; Luc, W.; Yan, Y.; Jiao, F.; Xu, B. Understanding Surface-Mediated Electrochemical Reactions: CO2 Reduction and Beyond. *8*, 8121–8129, Publisher: American Chemical Society.

(S4) Johnson, E. F.; Boutin, E.; Liu, S.; Haussener, S. Pathways to enhance electrochemical CO2 reduction identified through direct pore-level modeling. *1*, 704–719, Publisher: RSC.

(S5) Deng, W.; Zhang, P.; Seger, B.; Gong, J. Unraveling the rate-limiting step of two-electron transfer electrochemical reduction of carbon dioxide. *13*, 803, Number: 1 Publisher: Nature Publishing Group.

(S6) Ma, M.; Trześniewski, B. J.; Xie, J.; Smith, W. A. Selective and Efficient Reduction of Carbon Dioxide to Carbon Monoxide on Oxide-Derived Nanostructured Silver Electrocatalysts. *55*, 9748–9752, _eprint: https://onlinelibrary.wiley.com/doi/pdf/10.1002/anie.201604654.

(S7) Ma, M.; Liu, K.; Shen, J.; Kas, R.; Smith, W. A. In Situ Fabrication and Reactivation of Highly Selective and Stable Ag Catalysts for Electrochemical CO2 Conversion. *3*, 1301–1306, Publisher: American Chemical Society.

(S8) Luan, C.; Shao, Y.; Lu, Q.; Gao, S.; Huang, K.; Wu, H.; Yao, K. High-Performance Carbon Dioxide Electrocatalytic Reduction by Easily Fabricated Large-Scale Silver Nanowire Arrays. *10*, 17950–17956, Publisher: American Chemical Society.

(S9) Lu, Q.; Rosen, J.; Zhou, Y.; Hutchings, G. S.; Kimmel, Y. C.; Chen, J. G.; Jiao, F. A selective and efficient electrocatalyst for carbon dioxide reduction. *5*, 3242, Number: 1 Publisher: Nature Publishing Group.

(S10) Hsieh, Y.-C.; Senanayake, S. D.; Zhang, Y.; Xu, W.; Polyansky, D. E. Effect of Chloride Anions on the Synthesis and Enhanced Catalytic Activity of Silver Nanocoral Electrodes for CO2 Electroreduction. *5*, 5349–5356, Publisher: American Chemical Society.

(S11) Sun, K.; Wu, L.; Qin, W.; Zhou, J.; Hu, Y.; Jiang, Z.; Shen, B.; Wang, Z. Enhanced

electrochemical reduction of CO2 to CO on Ag electrocatalysts with increased unoccupied density of states. *4*, 12616–12623, Publisher: The Royal Society of Chemistry.

(S12) Zhang, L.; Wang, Z.; Mehio, N.; Jin, X.; Dai, S. Thickness- and Particle-Size-Dependent Electrochemical Reduction of Carbon Dioxide on Thin-Layer Porous Silver Electrodes. *9*, 428–432, _eprint: https://onlinelibrary.wiley.com/doi/pdf/10.1002/cssc.201501637.
